# DockStream: a docking wrapper to enhance de novo molecular design

**DOI:** 10.1186/s13321-021-00563-7

**Published:** 2021-11-17

**Authors:** Jeff Guo, Jon Paul Janet, Matthias R. Bauer, Eva Nittinger, Kathryn A. Giblin, Kostas Papadopoulos, Alexey Voronov, Atanas Patronov, Ola Engkvist, Christian Margreitter

**Affiliations:** 1 Molecular AI, Discovery Sciences, R&D, AstraZeneca, Gothenburg, Sweden; 2 Medicinal Chemistry, Research and Early Development, Cardiovascular, Renal and Metabolism (CVRM), BioPharmaceuticals R&D, AstraZeneca, Gothenburg, Sweden; 3 Structure & Biophysics, Discovery Sciences, R&D, AstraZeneca, Cambridge, UK; 4 Medicinal Chemistry, Research and Early Development, Respiratory and Immunology (R&I), BioPharmaceuticals R&D, AstraZeneca, Gothenburg, Sweden; 5 Medicinal Chemistry, Research and Early Development, Oncology R&D, AstraZeneca, Cambridge, UK; 6 Department of Computer Science and Engineering, Chalmers University of Technology, Gothenburg, Sweden

**Keywords:** De novo design, Generative Models, Reinforcement Learning (RL), Molecular docking, Structure-based drug discovery (SBDD)

## Abstract

Recently, we have released the de novo design platform REINVENT in version 2.0. This improved and extended iteration supports far more features and scoring function components, which allows bespoke and tailor-made protocols to maximize impact in small molecule drug discovery projects. A major obstacle of generative models is producing active compounds, in which predictive (QSAR) models have been applied to enrich target activity. However, QSAR models are inherently limited by their applicability domains. To overcome these limitations, we introduce a structure-based scoring component for REINVENT. DockStream is a flexible, stand-alone molecular docking wrapper that provides access to a collection of ligand embedders and docking backends. Using the benchmarking and analysis workflow provided in DockStream, execution and subsequent analysis of a variety of docking configurations can be automated. Docking algorithms vary greatly in performance depending on the target and the benchmarking and analysis workflow provides a streamlined solution to identifying productive docking configurations. We show that an informative docking configuration can inform the REINVENT agent to optimize towards improving docking scores using public data. With docking activated, REINVENT is able to retain key interactions in the binding site, discard molecules which do not fit the binding cavity, harness unused (sub-)pockets, and improve overall performance in the scaffold-hopping scenario. The code is freely available at https://github.com/MolecularAI/DockStream.

**Supplementary Information:**

The online version contains supplementary material available at 10.1186/s13321-021-00563-7.

## Introduction

Machine learning has emerged as a versatile tool with potential to accelerate drug discovery. One of the quintessential problems is de novo drug design which involves finding promising candidate molecules that satisfy a multi-parameter optimization (MPO) objective. [1, 2] The major obstacle is the sheer number of possible molecules, estimated to be on the order of 10 10^60 [23–60], effectively preventing a brute-force search of chemical space. [3] Recently, generative models have been proposed to sample chemical space beyond what is covered by established datasets by conferring the ability to sample novel compounds. Neural network architectures including recurrent neural networks (RNNs), variational autoencoders (VAEs), generative adversarial networks (GANs), and graph neural networks (GNNs) have demonstrated success in using input data as SMILES or molecular graphs to generate promising chemical ideas [2, 4–7]. Moreover, reinforcement learning (RL) has been applied in conjunction with generative models to apply an iterative design process in which an agent (a model) learns to generate compounds achieving increasing scores.

over time [4, 8, 9] RL encourages the agent to make decisions in order to maximize a reward function which can be tailored to optimize drug-like properties. The synergistic application of generative models and RL has demonstrated potential for de novo drug design by providing a solution to MPO and notably mitigating the computational burden of a brute-force search of chemical space. This approach is also very versatile in terms of components that can be optimized. Notably, quantitative structure–activity relationship (QSAR) models have been applied to great effect to enrich target activity [10]. However, such models are limited in their generalizability and thus often restricted to relatively small domains of applicability which hinders the ability to sample truly novel compounds [11, 12].

On the other hand, existing physics-based methods such as molecular docking continues to be an invaluable tool to identify molecules that are promising drug candidates [13–16]. The advent of high-performance computing (HPC) has enabled in silico virtual screening (VS) to consider increasingly larger datasets. This enhanced capability has seen success in identifying more hits with diverse chemotypes and scaffolds, often desirable in structure-based drug discovery (SBDD) [16]. VS typically screens molecular libraries whose compounds are readily available, offering the potential to expedite experimentation. However, chemical ideas are inherently restricted to the pre-defined chemical space of these collections which may not satisfy the rigorous criteria in a project. Instead, VS is often used to steer human creativity to a relevant chemical sub-space where iterative design discovers the final drug compound. While strategies such as screening privileged scaffolds can narrow the search space, computational costs of in silico docking rapidly becomes prohibitive and resources may be sub-optimally allocated towards exploring unproductive chemical space [17]. Thus, efficient traversal of chemical space remains a non-trivial endeavour.

More recently, molecular docking has been incorporated into RL generative model paradigms, offering a proposed solution that integrates structural information, steering molecular design by rewarding compounds that exhibit good docking scores and circumventing some limitations of QSAR models. [18–21] However, it is often challenging to ascertain what exactly constitutes a ‘good’ docking score. Docking algorithms are inherently sensitive to the three-dimensional (3D) representation of the protein and ligands [22, 23]. Moreover, different docking configurations may be better suited for particular targets, and performance is intricately dependent on ligand embeddings to sample sufficiently many conformations to access a binding pose deemed favourable. Consequently, while docking scores are a proxy for binding free energies, an accurate prediction is beyond the capability of docking algorithms, caused to a large extent by a lack of sampling of the receptor dynamics and insufficient treatment of entropic effects [24, 25]. Thus, in RL scenarios where docking is a component of the reward function, a poor choice of the docking algorithm or the docking score reward can accentuate the limitations of docking and misinform the agent.

Herein, we introduce DockStream, a molecular docking wrapper providing a unified interface to access a collection of ligand embedders and docking backends. DockStream provides an automated and streamlined platform to run docking experiments, supporting a large variety of possible configurations. Automated analysis of docking results expedites search for a docking configuration that performs best for the specific target and set of ligands—i.e. one that displays good correlation with experimental binding affinity or potency. The use of DockStream as a stand-alone tool for SBDD is demonstrated by benchmarking the DEKOIS 2.0 dataset which curates 81 targets with provided sets of active and decoy ligands [26]. The flexibility of DockStream to specify different docking configurations facilitates tailored protocols for different end applications as docking performance necessarily varies depending on the target system. Subsequently, DockStream is integrated with REINVENT 2.0, the recently published de novo design platform [4]. Docking provides structural information to the REINVENT agent, facilitating docking score optimization via RL and steering exploration to relevant chemical space. Using DockStream as a scoring function component enables REINVENT to design compounds that retain key interactions in the binding cavity while simultaneously discovering new ones.

## Application overview

### DockStream

**Fig. 1:**
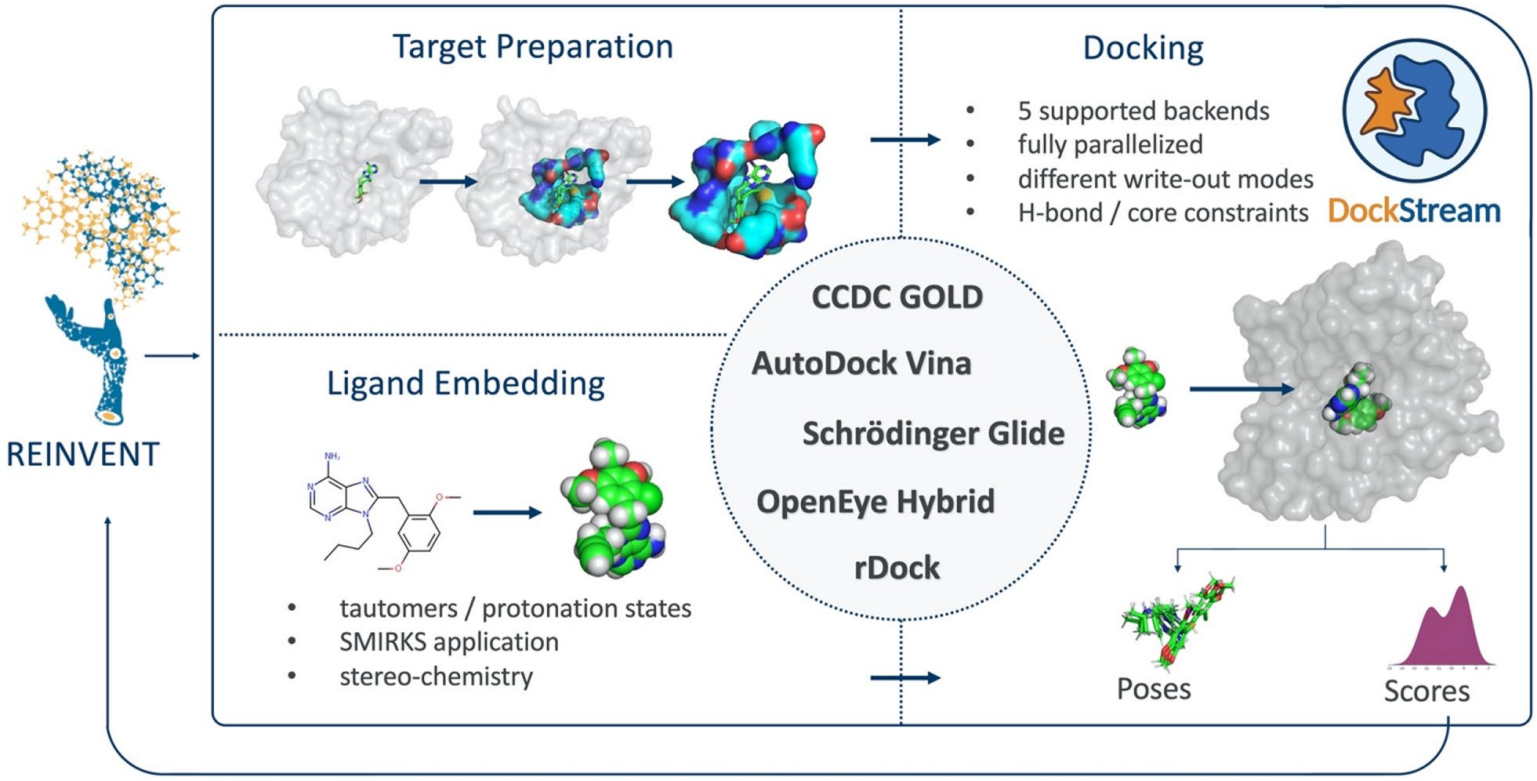
Overview of DockStream platform providing access to a variety of docking backends. Target preparation, ligand embedding, and docking are handled in the unified platform. The docking scores and poses are outputted

DockStream is a molecular docking wrapper providing access to a collection of ligand embedders: Corina, LigPrep, OMEGA, and RDKit, and docking backends: AutoDock Vina, Glide, GOLD, Hybrid, and rDock (Fig. 1) [27–41]. DockStream streamlines molecular docking by handling all necessary steps under a unified platform: target preparation, ligand embedding, and docking. Target preparation usually starts with refining a protein crystal structure, often involving adding missing hydrogen atoms, defining side chain ionization and tautomeric states, and minimizing the conformational energy [42]. The final orientation of amino acid residues around a reference ligand defines the binding cavity and the docking search space. Ligand embedding refers to the generation of 3D molecular configurations for the ligands that are to be docked, thus defining an initial ligand conformational state that can affect docking search space traversal [23]. By enumerating tautomeric states and stereoisomers, this stage can also lead to an expansion of the ligand set. Finally, docking itself adheres to specified parameters that control the rigor of a conformational search to generate so-called binding poses and scores that are typically used to inform SBDD. All of these preparatory steps must be considered in tandem to identify a useful docking configuration. DockStream provides numerous solutions to each component required for molecular docking via the supported backends whose parameters can be controlled by a single input JSON. The flexibility of the parameter definitions lends itself to applicability across diverse docking problems. Beyond providing access to a variety of backends, notable features of DockStream include:

1. Ligand tautomer/stereoisomer handling providing a thorough enumeration of atom spatial arrangements and states.
2. Different write-out modes to output poses and scores corresponding to either the best per ligand (and all its enumerations), best per enumeration (each tautomer/stereoisomer generated), or all ligand enumerations.
3. Parallelization across cores to speed-up execution (see Additional file 1: Fig. S1).

An example LigPrep configuration in an input JSON is shown below (see Additional file 1 for an example full input JSON that includes docking) [30]:
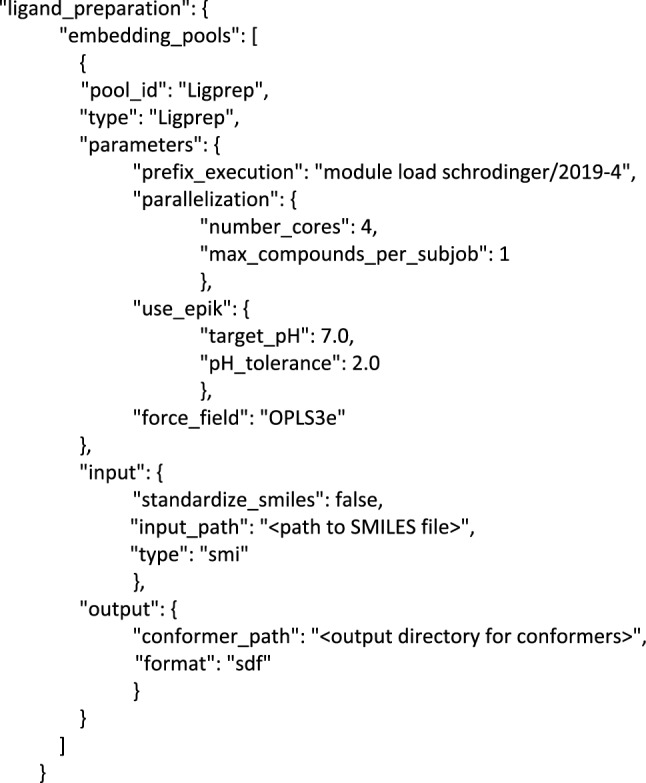


Notable settings include:

1. The “parallelization” block specifies to parallelize LigPrep over 4 CPU cores, distributing a maximum of 1 compound per core at a time.
2. The “use_epik” block provides access to LigPrep capabilities including specifying a desired pH and a pH tolerance.
3. The “force_field” block allows one to specify the desired force field which may be particularly relevant when reproducing previous docking experiments.
4. The “input” block specifies the input path for the SMILES to be embedded by LigPrep.
5. The “output” block is optional and specifies the desired output directory for the embedded ligands.

Once an input JSON is constructed, DockStream can be excecuted via the command line:

python docker.py -conf < path to input JSON > 

### Docking evaluation

**Fig. 2:**
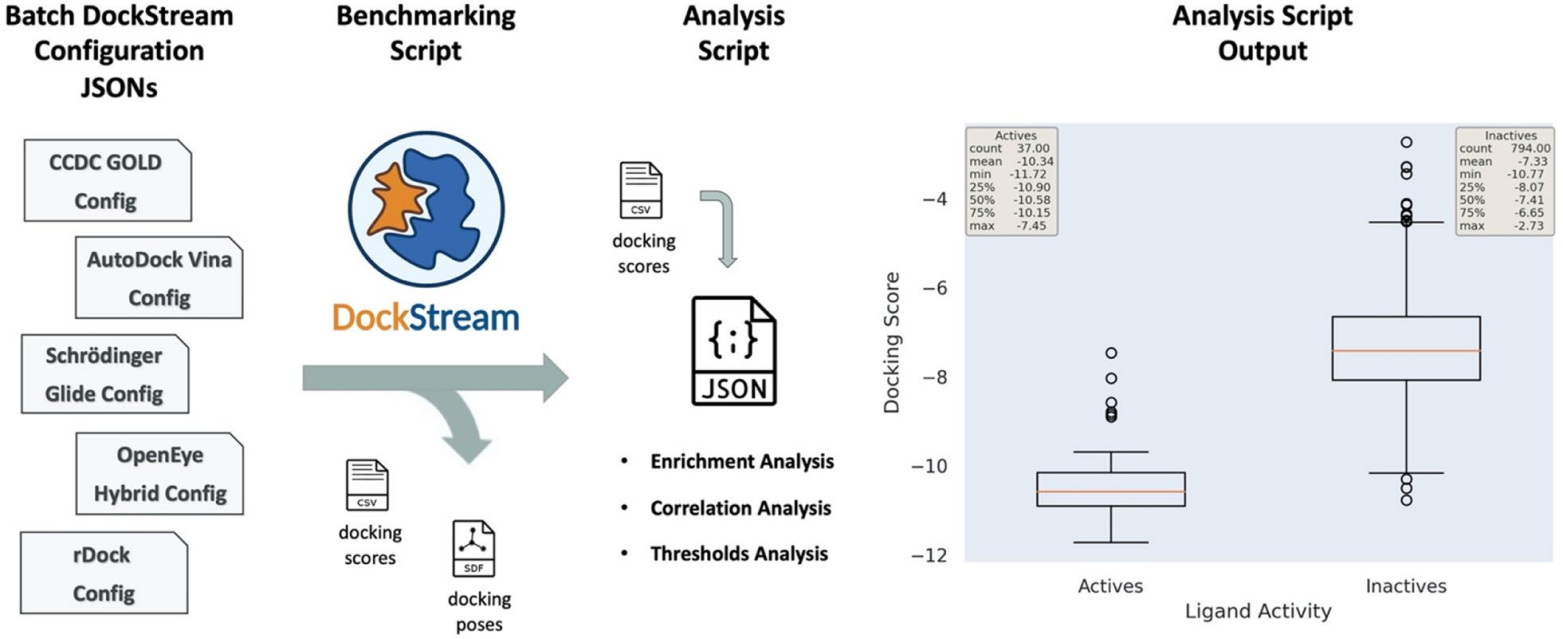
DockStream automated molecular docking workflow via the benchmarking and analysis scripts. Each configuration JSON file specifies all the parameters required for a docking experiment. The analysis script supports 3 analysis modes: Enrichment, Correlation, and Thresholds (see Additional file 1 for details)

The suitability of an in silico docking configuration is usually assessed by its sampling or scoring power [23]. The former refers to the ability to reproduce binding poses of reference ligand-receptor co-crystal structures, often measured by the root-mean-square deviation (RMSD) of the atomic coordinates. The latter refers to the ability to rank binding affinities based on the docking score. In the ideal scenario, the docking configuration displays excellent sampling and scoring power, but this is typically not observed. Rather, docking algorithms may be better suited for either task where choice of a docking configuration will be dependent on the desired use case [39, 43]. In practice, finding an informative docking configuration is challenging owing to the inherent sensitivity of scoring functions to the 3D representation of proteins and ligands. DockStream expedites this search by automating the execution and analysis of molecular docking experiments via the benchmarking and analysis scripts, respectively (Fig. 2).

### Benchmarking

Using DockStream, a single input JSON file defines all parameters required for a molecular docking experiment. Modifying parameters in the JSON defines a new docking configuration and offers complete flexibility in executing all supported backends. The benchmarking script in turn takes as input an arbitrary number of configuration JSONs, and executes all defined docking experiments sequentially, returning the docked poses and scores (Fig. 2). Analysis of resulting docking scores can be automated by the analysis script which supports three modes (see Additional file 1 for details regarding each mode):

1. Enrichment analysis to evaluate whether a docking configuration scores active ligands better than decoys (on average).
2. Correlation analysis to evaluate how well, if at all, the docking scores correlate with experimental results.
3. Thresholds analysis to probe the distribution of docking scores and experimental results given a set of defined thresholds that serve as a hard boundary between classification as active or inactive. This functionality is well suited to investigate general separation of data points in cases where poor correlation between docking scores and experimental results is observed.

Similar to docking, a single input JSON defines all parameters required for the analysis script. Moreover, all analysis modes take as input an arbitrary number of docking outputs. Therefore, the DockStream benchmarking and analysis scripts workflow is pertinent to expedite search for a docking configuration well suited for various SBDD applications.

### Enrichment analysis

The ability of a docking configuration to distinguish between active and decoy ligands is useful for hit discovery where the goal is to identify a sufficiently active ligand against a target. The logarithmic receiver operating characteristic (pROC) area under the curve (AUC) is calculated and provides a measure of enrichment. The logarithmic transform of the classical ROC curve biases the contributions to the AUC towards early actives recovered and is therefore particularly advantageous for evaluating early enrichment [44]. The pROC AUC is given by Eq. 1:

1 $$pROC AUC = \frac{1}{n}\mathop \sum \limits_{i}^{n} log_{10} \left( {\frac{1}{{\beta_{i} }}} \right)$$

where $n$ is the total number of active and decoy ligands and ${\beta }_{i}$ is the false positive rate (FPR) for when the $i$ th active is recovered in the ordered (by docking score) list. A greater pROC AUC value demonstrates enrichment and is attributed to the ability of a docking configuration to distinguish between actives and decoys. The pROC AUC expected for random selection is 0.434 (in contrast to 0.5 for the classical ROC AUC). Importantly, unlike the ROC AUC that enforces an upper bound of 1.0, the pROC AUC is formally unbounded. In practice, however, it is constrained by the relative frequency of recovering actives amongst decoys based on HTS [44].

### Correlation analysis

In lead optimization, one may be interested in increasing potency. Docking scores are often used as a crude proxy for binding free energies and thus, it would be straightforward to simply consider compounds exhibiting good scores. However, accurate prediction of binding free energies is often beyond the scope of these scoring functions and there is no guarantee the aforementioned approach will yield novel leads with enhanced potency [24, 25]. Instead, identifying a docking configuration that displays good correlation between docking scores and experimental binding affinities for a calibration set can guide iterative lead compound selection and bolster confidence in the results. The Spearman (ρ) ∈ $\left[ { - 1, 1} \right]$ and Kendall Tau-b ($\tau_{B}$) ∈ $\left[ { - 1, 1} \right]$ rank correlation coefficients provide a quantitative measure between ordinal variables [45, 46]. The Spearman correlation is given in Eq. 2:

2 $$\rho = 1 - \frac{{6\mathop \sum \nolimits_{i = 1}^{n} d_{i}^{2} }}{{n\left( {n^{2} - 1} \right)}}$$

where $n$ is the total number of ligands and $d_{i}$ is the difference between the ranks of the $i$ th docking score and experimental binding affinity. If the docking scores and experimental binding affinities are perfectly monotonic with respect to each other, then $\rho = 1$ (see Additional file 1 for details). The Kendall Tau-b correlation is given in Eq. 3:

3 $$\tau_{B} = \frac{C - D}{{\sqrt {\left( {C + D + T_{dock} } \right)*\left( {C + D + T_{exp} } \right)} }}$$

where $C$ and $D$ are concordant and discordant pairs, respectively, and $T_{dock}$ and $T_{exp}$ are the number of ties in the docking scores and experimental binding affinities data, respectively. Similar to Spearman, if the data is perfectly monotonic with respect to each other, then $\tau_{B} = 1$. An important distinction between Spearman and Kendall is that the latter accounts for ties in the data [46]. This is particularly relevant in binding assays where compound activities may exceed the limit of quantification (LOQ). Consequently, these compounds are assigned the value at the limit, resulting in ties in the data. In general, Kendall is a more informative measure than Spearman in these cases.

### REINVENT overview

REINVENT is a de novo design platform that uses generative models to sample compounds in SMILES format. [4, 47] SMILES generation is formulated as a natural language processing (NLP) problem whereby atoms are tokenized into machine-readable vocabulary. Compound SMILES are then generated token by token based on sequential conditional probabilities, analogous to a typical NLP problem. The underlying architecture of REINVENT uses RNNs and is based on work by Arús-Pous et al. [48]. The specific architecture used in this work is a RNN with embedding size 256, three hidden layers of 512 gated recurrent unit (GRU) cells, and a linear layer with softmax activation [49]. Model training followed two steps: first, a prior generative model was trained on the ChEMBL dataset [50]. The agent policy was initialized based on the prior before diverging into individual experiments. The RL was conducted for 1000 epochs, thus iteratively calibrating the agent’s likelihood of sampling desirable compounds (see Additional file 1 for details).

### Scoring function

The scoring function, $S\left( x \right)$ ∈ $\left[ {0, 1} \right]$ offers complete flexibility in the defined properties to optimize, including topological polar surface area (TPSA), molecular weight (MW), number of hydrogen bond donors (HBD), molecular docking score, and custom QSAR models. In this work, $S\left( x \right)$ is formulated as a weighted geometric mean given in Eq. 4:

4 $$S\left( x \right) = \left( {\mathop \prod \limits_{i = 1}^{n} P_{i} \left( x \right)^{{w_{i} }} } \right)^{{1/\mathop \sum \limits_{i = 1}^{n} w_{i} }}$$

where $n$ is the number of properties, $P_{i} \left( x \right)^{{w_{i} }}$ is the score calculated for the $i$ th property for the sampled compound, and $w_{i}$ is the weighting for the $i$ th property. Properties assigned a greater weighting contribute more significantly to $S\left( x \right)$ and the weighted geometric mean necessitates every property to be reasonably satisfied. Otherwise, the total score diminishes eliciting negligible change during agent feedback. In this work, all properties were assigned a weighting of 1, denoting equal importance. Greater weightings such as 5 or 10 may be assigned to target properties to increase their relative importance in the scoring function. For instance, given a lead optimization task to enhance predicted potency and solubility, one could assign a greater weighting to docking and the corresponding QSAR model.

### Diversity filters (DF)

A consequence of the iterative design cycle is that the agent may become stuck in local minima and sample increasingly similar compounds so as to exploit a single solution found. A diversity filter (DF) is applied to penalize sampled compounds based on the notion of bucket saturation (details as in the REINVENT 2.0 paper) [4, 47]. The topological DF is used in this work which takes a compound scaffold and converts all atoms into sp3 carbons. Compounds with similar scaffolds that satisfy $S\left(x\right)$ are stored in buckets with limited size (25 in this work). Buckets are filled when such compounds are repeatedly sampled. Once full, the next compound with scaffold similarity above a certain threshold will penalize the agent to disincentivize further sampling of the scaffold and encourage exploration to other chemical space. This prevents mode collapse and ensures a diverse library of generated compounds [51].

### REINVENT-DockStream integration

DockStream can be specified as a component to REINVENT (see Additional file 1: Fig. S15 for more details), conferring the ability to incorporate molecular docking into $S\left(x\right)$. While it is straightforward to impose docking score optimization in REINVENT, defining a useful docking configuration remains challenging. Ideally, the docking score should be biased to either exhibit high correlation with experimental activity or at least be able to distinguish active and decoy ligands. Similar to using DockStream as a stand-alone docking tool, the benchmarking and analysis workflow (Fig. 2) can be used to automate search for such a docking configuration. There are two use cases:

1. Enrichment analysis to assess the ability of the docking configuration to distinguish between active and decoy ligands. Here, the range of docking scores for active ligands is important to define an appropriate transformation function, $P$ ∈ $[0, 1]$, that normalizes the raw score such that it can be integrated into $S\left(x\right)$. Ultimately, the agent learns to propose compounds that score well using the given docking configuration.
2. Correlation analysis to assess the ability of the docking configuration to rank compounds relative to their experimental activity. Here, higher values for the correlation metrics are desired with attention given to experimental assays that artificially restrict the range of values due to the LOQ. Compounds that score well should have a greater likelihood of being potent binders.

Identifying an informative docking configuration enhances the quality of the compounds proposed by REINVENT. In scaffold hopping scenarios with docking, sampled compounds are able to retain key interactions while simultaneously exploring new ones. As long as the binding pose is similar to that of known binders, this approach can guide the agent to produce promising candidate molecules. Importantly, this can dissuade entropically driven binding which is notorious for off-target activity and toxicity, provided the generated ligand maintains similar lipophilic ligand efficiency [52].

## Results

### DockStream DEKOIS 2.0 evaluation

**Fig. 3:**
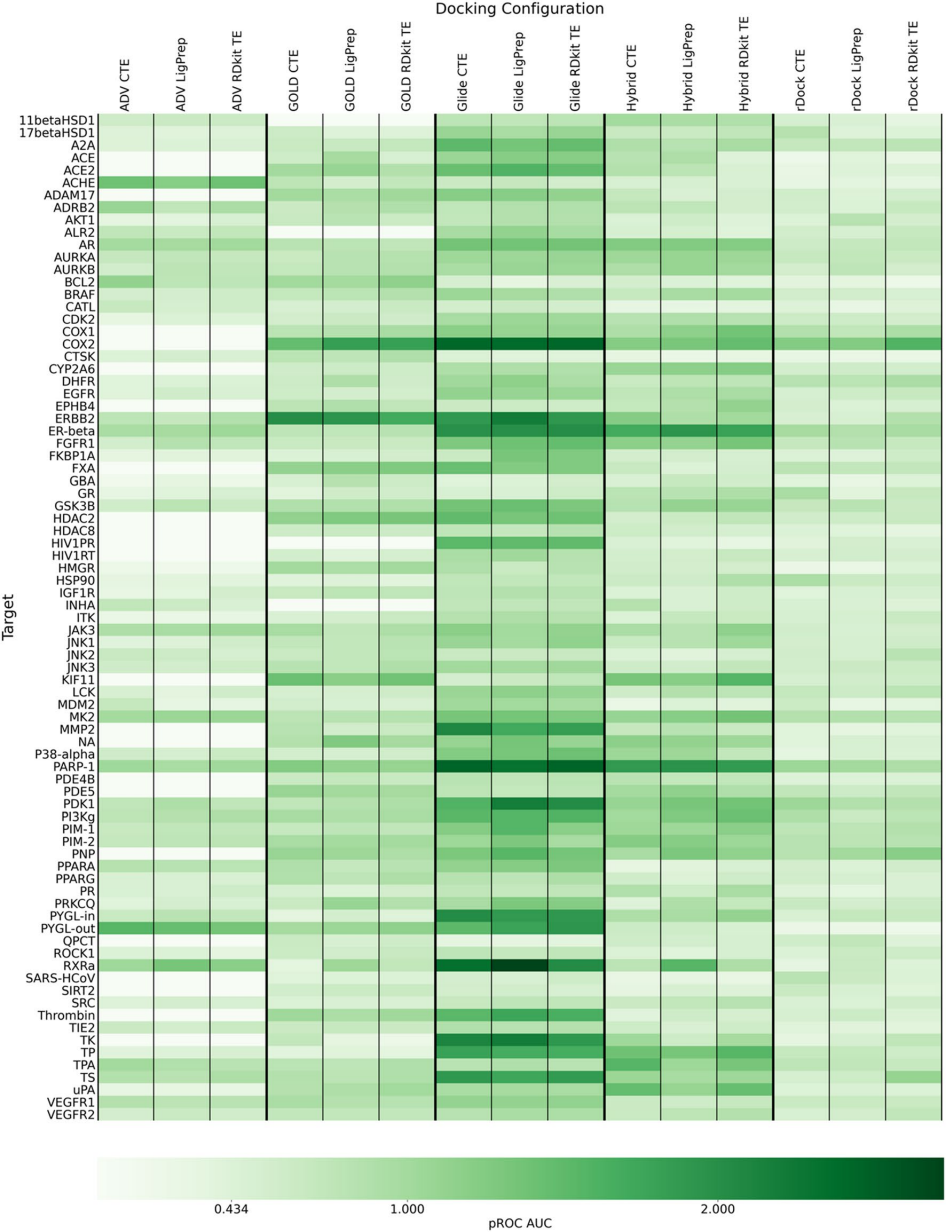
DockStream DEKOIS 2.0 benchmarking.^26^ Docking configurations used represent every combination possible between the docking backends: AutoDock Vina (ADV), GOLD, Glide, Hybrid, and rDock, and the ligand embedders: Corina, LigPrep, and RDKit. CTE is Corina with TautEnum and RDKit TE is RDKit with TautEnum. See Methods for parameters used. The pROC AUC of a docking configuration that is no better than random selection in distinguishing active and decoy ligands is 0.434. A pROC AUC greater than 0.434 denotes enrichment

The use of DockStream as a stand-alone tool was demonstrated by benchmarking the DEKOIS 2.0 dataset which curates 81 targets each with 40 actives and 1200 decoys. The dataset was curated to represent a reasonable fair assessment of docking performance by addressing diverse properties, including removal of Pan Assay Interference Compounds (PAINS) and covalent binders in bioactivity data, ensuring similar physico-chemical property matching between actives and decoys, and ensuring diverse chemotypes to avoid scaffold bias [26, 53]. The overall docking results reproduce and extend the observations of the original DEKOIS 2.0 benchmarking. The 5 supported docking backends: AutoDock Vina, GOLD, Glide, Hybrid, and rDock were paired with 3 ligand embedders: Corina with TautEnum, LigPrep, and RDKit with TautEnum to generate 15 distinct docking configurations [27–30, 33–38, 40, 41, 54, 55]. It is important to note that each docking configuration has many tunable parameters and screening every permutation is infeasible. Therefore, the default settings were kept with a few exceptions (see Methods for details). The benchmarking script was used to automate docking using each docking configuration for all 81 targets, resulting in 1215 docking runs and over 1,500,000 ligands docked. The analysis script was used to automate enrichment analysis and the pROC AUC values are shown in Fig. 3. The pROC AUC for a docking configuration that is no better than random selection in distinguishing actives and decoys is 0.434. Glide led to the most observed enrichment followed by Hybrid and GOLD, while the open-source docking backends, AutoDock Vina and rDock yielded less overall enrichment. In some cases, AutoDock Vina and GOLD failed to dock the entire set of ligands (see “Methods” section for details). In general, docking backends are not very sensitive to the choice of ligand embedder for the DEKOIS 2.0 targets. However, there are cases where the ligand embedder does have a significant effect on observed enrichment. For instance, AutoDock Vina with Corina and TautEnum for BCL2, Hybrid with LigPrep for RXRa, and rDock with RDKit and TautEnum for COX2, perform best within their ligand embedder series. Therefore, no single ligand embedder always performs best and while screening through $n$ different ligand embedders necessitate $n$ docking runs, increases in enrichment may identify a valuable docking configuration that otherwise would have been overlooked. Furthermore, no single docking backend performed best for all targets (compare COX2 Glide and AutoDock Vina ACHE for example). Alternatively, the goal may be to identify a reasonably good docking configuration with the highest throughput. Hybrid is particularly well suited for this owing to its speed and it may be the case that it also performs best or close to best performing as observed in the case of KIF11 and AR, respectively. Naturally, docking varies in performance for different target systems and ligand sets. The flexibility and ease of specifying different docking configurations and automating their execution and analysis demonstrates the value of a tool like DockStream.

### REINVENT-DockStream experiments

REINVENT experiments were performed with DockStream as a specified component to demonstrate the agent can learn to optimize docking scores and hence implicitly incorporate 3D structural information. To enforce “drug-likeliness”, the MPO problem was defined as optimizing the following properties with resemblance to Lipinski’s Rule of 5 [56]:

1. Docking score (docking backend and target specific, see Additional file 1: Figs. S17–S33)
2. Quantitative Estimate of “Druglikeness” (QED) Score ∈ $[0, 1]$ [57]
3. Number of hydrogen bond donors (HBD) ∈ $[0, 7]$ (see Additional file 1: Fig. S16a)
4. Molecular weight (MW) ∈ $[200, 575]$ (see Additional file 1: Fig. S16b)

In our experience, enforcing QED optimization made the molecular generation process more challenging but was necessary for the agent to propose “drug-like” compounds [58]. In the absence of QED, the agent learns to exploit the weak spots of docking scoring functions, proposing unreasonable molecules with artificially good docking scores. Furthermore, a well known phenomenon in RL is catastrophic forgetting in which the agent forgets previous learnings over the course of training [59]. To mitigate this, inception is used which is based on experience replay whereby top scoring compounds are randomly replayed to the agent during training and is implemented as described by Blaschke et al. [4].

**Fig. 4:**
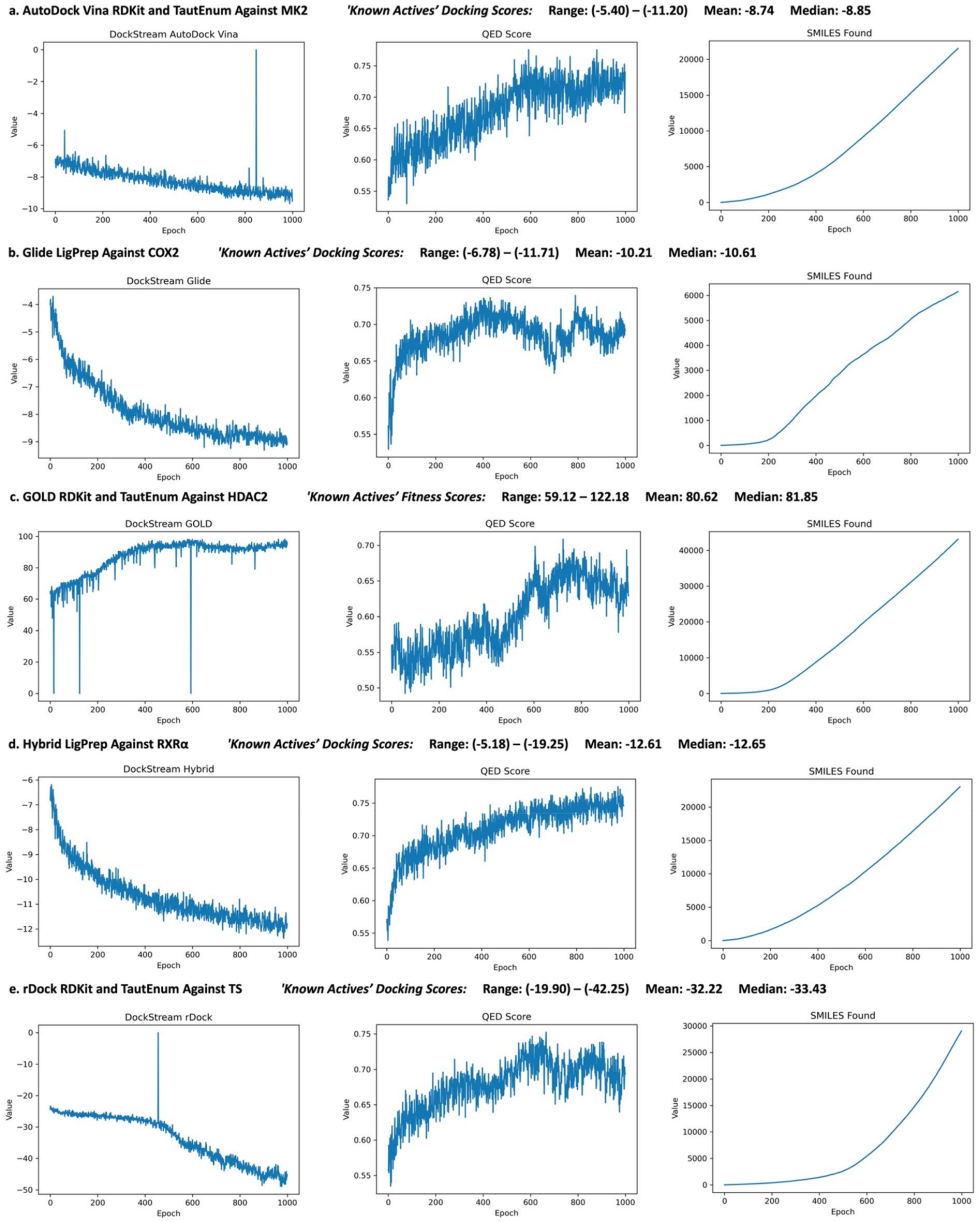
REINVENT-DockStream agent reinforcement learning training progress for selected experiments: **a** AutoDock Vina with RDKit and TautEnum against MK2 (**PDB ID: 3KC3**), **b** Glide with LigPrep against COX2 (**PDB ID: 1CX2**), **c** GOLD with RDKit and TautEnum against HDAC2 (**PDB ID: 3MAX**), **d** Hybrid with LigPrep against RXRα (**PDB ID: 2P1RT**), and **e** rDock with RDKit and TautEnum against TS (**PDB ID: 1I00**) (see Additional file 1: Figs. S16–33 for training plots of all experiments). ‘Known Actives’ docking scores (fitness scores for GOLD) from the DEKOIS 2.0 dataset are shown.^26^ Docking and QED score optimization and the number of SMILES found are shown. Each epoch proposes batch size (128) number of compounds. Lower docking scores for AutoDock Vina, Glide, Hybrid, and rDock, and higher fitness scores for GOLD are considered better. The direction of the docking score optimizations reflect this difference. ‘SMILES found’ refers to the cumulative number of unique compounds proposed that pass a total score threshold. If every epoch generates only unique, valid, and favourable compounds, the plot is linear

REINVENT-DockStream experiments were performed for all 15 backend and ligand embedder combinations used in the DEKOIS 2.0 benchmarking (Fig. 3). 15 targets were selected (1 for each backend and ligand embedder combination) based on exhibiting a pROC AUC > 1, indicating actives scoring better than decoys (on average) (see Additional file 1: Table S1 for details). The rationale was that given the agent learns to optimize the docking scores, selecting an appropriate docking score transformation will steer the REINVENT agent to propose compounds that are more likely to be true binders. Sampled training plots (1 for each docking backend) for the REINVENT-DockStream experiments are shown in Fig. 4 (see Additional file 1: Figs. S16–33 for all plots). The agent learned to propose compounds with increasingly favourable docking scores for every docking backend. In the case of AutoDock Vina, GOLD, and rDock (Fig. 4a, c, and e), the docking scores learning curves fluctuate and sometimes exhibit sharp spikes, in contrast to the smooth optimizations observed for Hybrid and Glide (Fig. 4b and d). The difference in agent performance is attributed to the stochasticity of AutoDock Vina, GOLD, and rDock where compounds proposed at select epochs can be notably worse while docking scores continue to improve, on average [33, 38, 41]. The sharp spikes correspond to the agent exploring chemical space as enforced by the DF and in which generated compounds fail to dock or dock poorly. While changing the docking parameters such as removing constraints or increasing sampling time can make docking more robust to these events, the momentary drop in performance did not hinder overall optimization across all docking backends. At the end of the 1000 epochs, the docking scores for all experiments converged to a point where the average score for the batch of compounds proposed by the agent are in the same range as the active ligands provided in the DEKOIS 2.0 dataset. Moreover, the top compounds (based on total score) display even more favourable docking scores, especially when $S(x)$ uses a weighted geometric mean, necessitating every property to be reasonably optimized. Thus, the top proposed compounds at the very least exhibit docking scores that are similar, if not better, than verified active ligands. The QED scores were also improved on average for every docking backend, ensuring proposed compounds are “drug-like” (Fig. 4). Similar to docking, momentary drops in QED optimization can be attributed to the stochasticity of agent exploration. Other properties that were optimized include the number of hydrogen bond donors and molecular weight (see Additional file 1: Figs. S16–33). The transformed scores for these properties across all docking backends and across all epochs ranged between 0.77 and 0.99, as expected since the prior was trained on ChEMBL which curates “drug-like” compounds (see Additional file 1: Figs. S16–S3). While most compounds in ChEMBL are Lipinski compliant, the rules are simply a guideline and there are many drug molecules that violate it [56]. The rationale in enforcing the number of hydrogen bond donors (increased to 7) and molecular weight (200–575 Da) was that keeping proposed compounds within this expanded guideline should increase the likelihood of obtaining a successful first candidate molecule when domain knowledge is typically not well established. It follows that docking scores are generally more meaningful amongst Lipinski compliant compounds, preventing molecules from being completely decorated with -OH groups or possessing excessive hydrophobicity that exploits the docking algorithm to achieve an artificially high docking score. Furthermore, the number of unique SMILES found exhibit almost a linear relationship for all docking backends (Fig. 4) indicating agent exploration. The exception was rDock which generated less favourable and unique compounds at the start of the REINVENT experiment (Fig. 4e) which is attributed to the agent exploring similar and unfavourable chemical space at the beginning of training. Importantly, agent training is demonstrated as all properties including docking were still optimized over time. The percentage of valid SMILES for all experiments and across all epochs was between 90 and 99% which was also expected as the prior was trained on ChEMBL (see Additional file 1: Figs. S17–33). The outcome of the REINVENT-DockStream experiments are libraries of proposed compounds that are drug-like and exhibit docking scores similar or better than verified active ligands.

### Agent exploration and exploitation

**Fig. 5:**
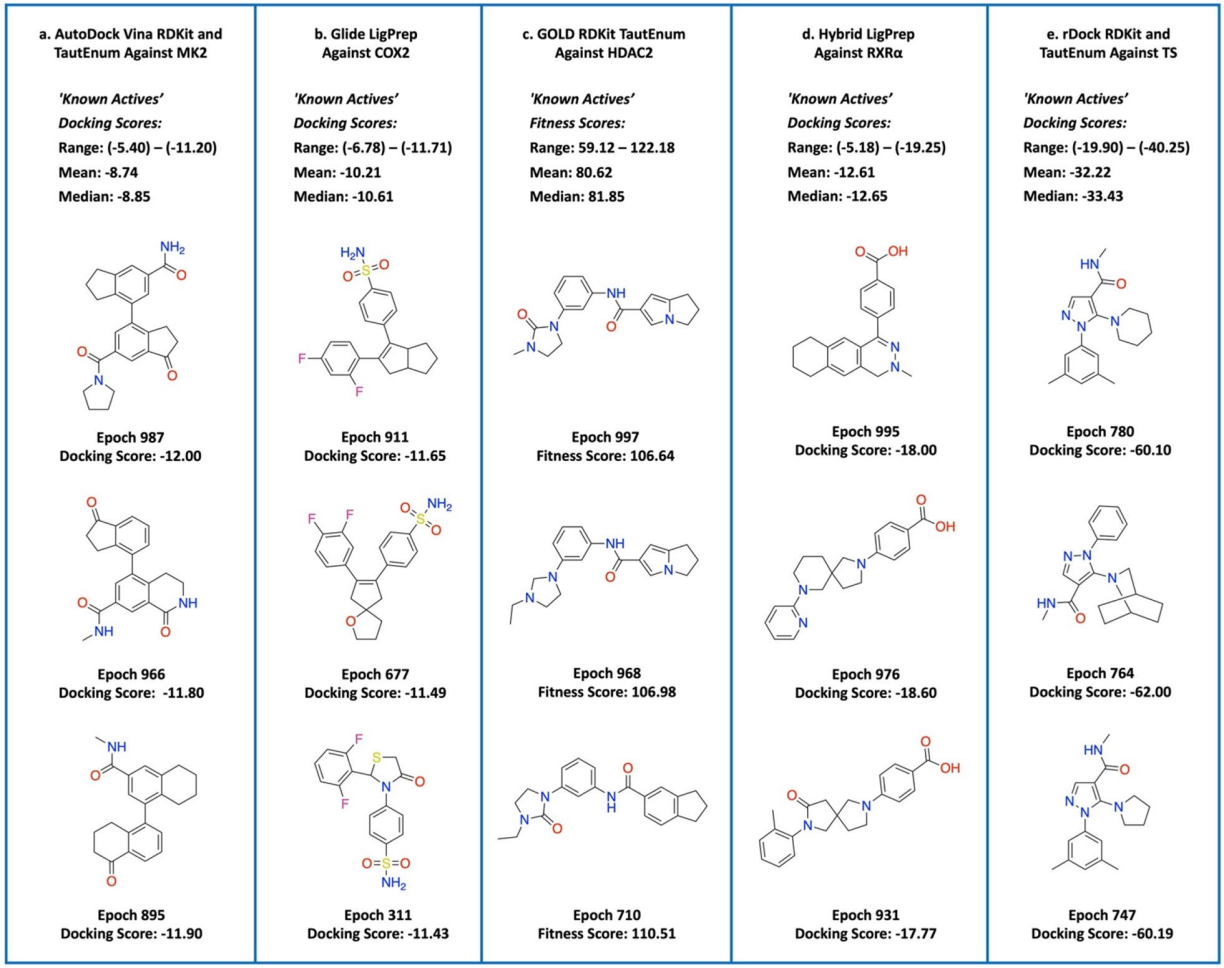
Selected top compounds generated from the experiments shown in Fig. 4. Epoch refers to the cycle in which they were observed, and corresponding docking scores (fitness scores for GOLD) are shown. **a** AutoDock Vina with RDKit and TautEnum against MK2 (**PDB ID: 3KC3**), **b** Glide with LigPrep against COX2 (**PDB ID: 1CX2**), **c** GOLD with RDKit and TautEnum against HDAC2 (**PDB ID: 3MAX**), **d** Hybrid with LigPrep against RXRα (**PDB ID: 2P1T**), and **e.** rDock with RDKit and TautEnum against TS (**PDB ID: 1I00**). ‘Known Actives’ docking scores (fitness scores for GOLD) from the DEKOIS 2.0 dataset are shown. [26]

Compound diversity is exemplified in Fig. 5 which displays 3 out of the top 10 compounds (based on total score) of each REINVENT-DockStream experiment. In general, the top compounds were generated at later epochs and possess diverse scaffolds from sampling numerous local minima, effectively circumventing mode collapse [51]. Agent exploration is demonstrated by both the training plots which display gradual optimization of docking scores (Fig. 4) and the top sampled compounds (Fig. 5) which feature diverse scaffolds. Furthermore, agent perceived desirable scaffolds can be generated multiple times, as illustrated by GOLD and rDock (Fig. 5c and e). Interestingly, the top GOLD and rDock compounds shown in Fig. 5c and e share subtle differences and vary only by 1 or 2 atoms. The scores achieved by these compounds were nearly identical and suggests the agent implicitly learns 3D structural information when docking is incorporated into the scoring function. This is further exemplified by the Glide docking experiments performed against cyclooxygenase 2 (COX2) which is a target for anti-inflammation (Fig. 5b) [60, 61]. The proposed sulfonamide moiety (also observed ubiquitously in replicate experiments) is present in the approved drugs, Celecoxib and Valdecoxib, although the latter was discontinued due to cardiovascular toxicity [61]. The agent was initialized based on a random sampling of the ChEMBL dataset and therefore did not possess any preconceived structural bias. The generation of sulfonamide containing compounds suggests agent structural awareness capable of exploiting the shape and electrostatics of the binding cavity. Overall, the top compounds (based on total score) across all experiments contained compounds that optimized every component specified in the scoring function. The general diversity observed for the top proposed compounds provide multiple solutions to the MPO problem and demonstrates the synergistic application of agent exploration with a DF enforced.

Compounds generated at similar epochs can share the same scaffold, as exemplified by the GOLD (epochs 997 and 968) and rDock (epochs 780 and 747) top selected compounds (Fig. 5c and e).

Intuitively, agent generated compounds from neighbouring epochs are expected to share a greater similarity than epochs further apart, as the agent is sampling from an area “close-by” in chemical space. Also, one would expect this trend to be more pronounced the more focused the agent gets over the course of the training. In order to quantify this behaviour, the average linkage similarity (Tanimoto) between every 5 epochs has been calculated (see “Methods” section for details) for every REINVENT-DockStream experiment (See Additional file 1: Figs. S35–51) [62] Fig. 6b shows the resulting Tanimoto matrix for the GOLD experiment displayed in Figs. 4c and 5c. Firstly, the main diagonal of the Tanimoto matrix.

**Fig. 6:**
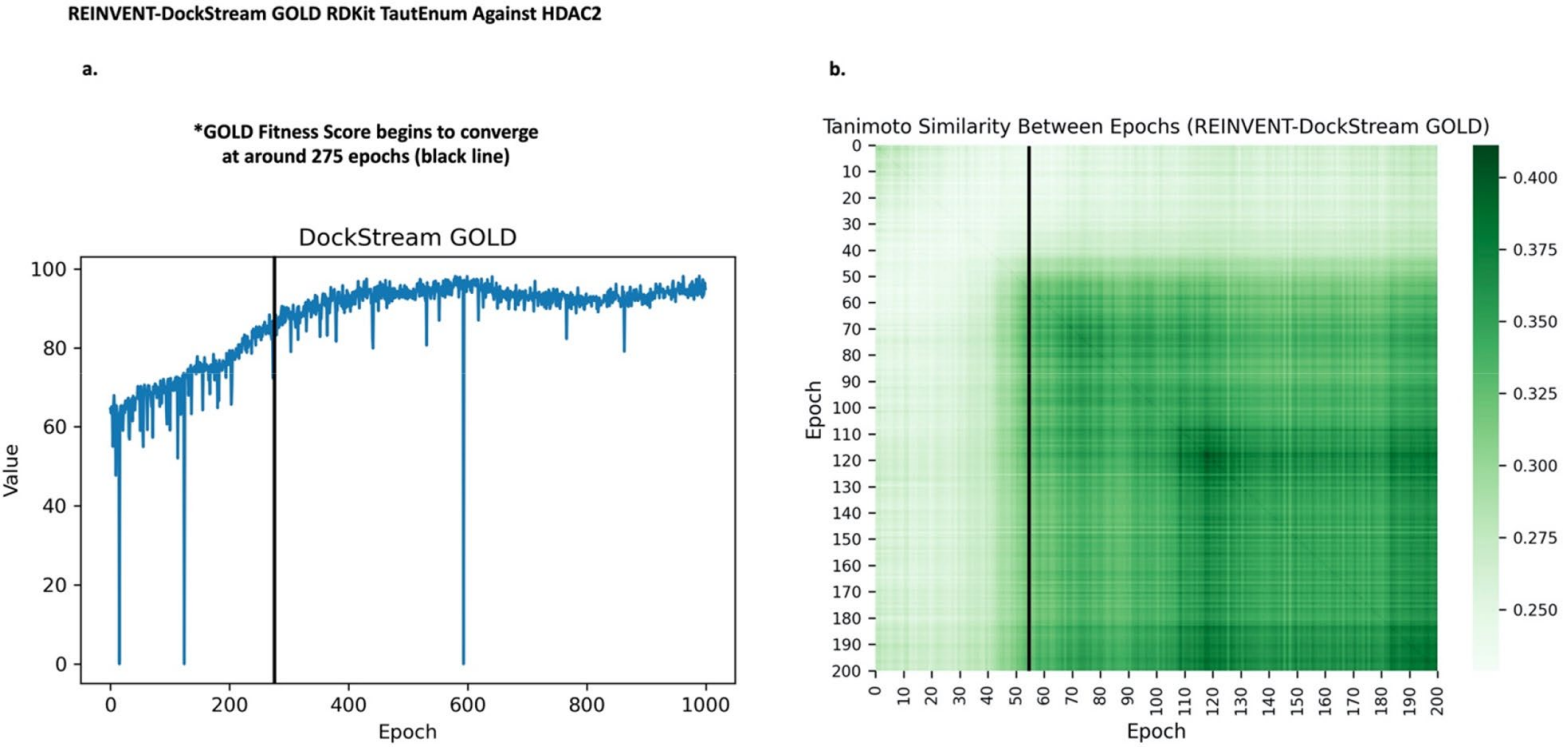
Average linkage similarity between epochs (Tanimoto) for every 5 epochs for REINVENT-DockStream using GOLD with RDKit and TautEnum against HDAC2 (**PDB ID: 3MAX**). **a** GOLD fitness score training plot (same as Fig. 4c). The vertical black line at around epoch 275 indicates the start of convergence whereby the GOLD fitness score begins to plateau. **b** GOLD Tanimoto matrix illustrating the Tanimoto similarities between batches of generated compounds across the entire 1000 epochs REINVENT-DockStream experiment (x-axis is on the scale of 5 epochs, e.g. epoch 10 and 200 correspond to epoch 50 and 1000, respectively). The main diagonal is darker shaded, indicating notable intra-batch compound similarity. The overall matrix transitions from lighter (top left) to darker (bottom right) shaded areas. Cross-referencing with subplot **a**, neighbouring epochs display notably greater Tanimoto similarity, coinciding with GOLD fitness Score convergence. The results suggest the agent begins exploitation once a state of productivity is achieved (as measured by fitness score convergence). The overall transition of the matrix demonstrates agent exploration and exploitation is darker (indicating higher similarity) relative to surrounding epochs and gradually becomes even darker, which indicates increased intra-batch similarity as the agent increasingly focuses on regions in chemical space. Moreover, the transition between the lighter shaded top left corner to the darker shaded bottom right corner exemplifies balance between agent exploration and exploitation. By cross-referencing the REINVENT-DockStream training plot for GOLD docking (Fig. 6a), one can identify that the GOLD docking score begins to converge at around epoch 275. At around epoch 55 in Fig. 6b (corresponds to epoch 275), the Tanimoto matrix gradually becomes darker shaded, indicating increased Tanimoto similarity within the same batch and neighbouring epochs batches (Fig. 6b). The results demonstrate policy update, reaching a state of productivity and enforcing the agent to begin exploitation of chemical.

space. Furthermore, even once exploitation begins, the Tanimoto similarities do not converge to 1. The agent continues to explore chemical space as necessitated by the DF applied. In addition, one can observe that the Tanimoto similarities corresponding to surrounding epochs is greater (on average), further supporting gradual and iterative policy update. The results show that REINVENT achieves both chemical space exploration and exploitation and enforces sampling of numerous local minima.

### Comparison of generated compounds to known actives and decoys

**Fig. 7:**
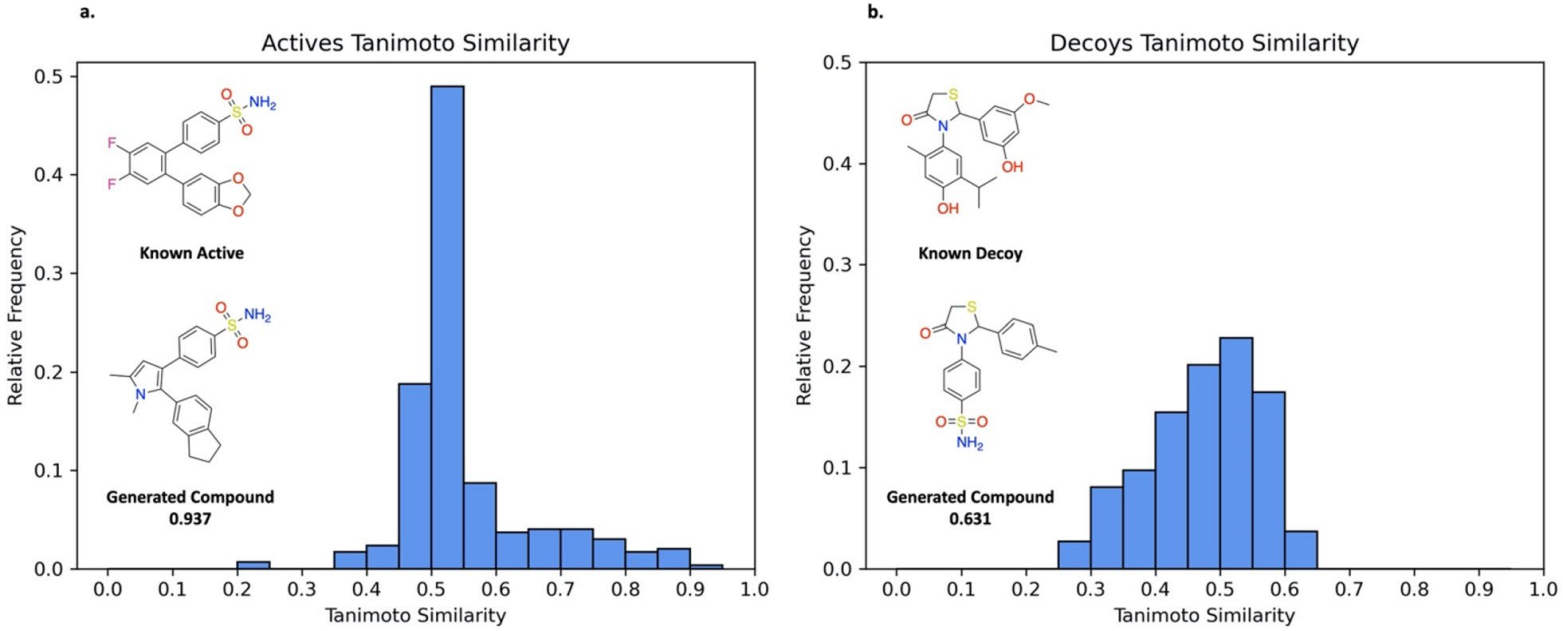
Distributions of the highest Tanimoto similarities for the top compounds compared to known actives and decoys provided in the DEKOIS 2.0 dataset [26]. REINVENT-DockStream using Glide with LigPrep against COX2 (**PDB ID: 1CX2**) was ran in triplicate. The top 150 compounds from each experiment were pooled and duplicates removed, resulting in 298 unique top compounds. Each of these compounds were compared to each active (40 total) or decoy (1200 total) and the highest Tanimoto similarity kept. **a** Highest Tanimoto similarity analysis for the COX2 actives. The most similar compound achieved a Tanimoto similarity of 0.937 compared to the ‘Known Active’. **b** Highest Tanimoto similarity analysis for the COX2 decoys. The most similar compound achieved a Tanimoto similarity of 0.631 to the ‘Known Decoy’

As an initial investigation into the quality of generated compounds, REINVENT-DockStream using Glide with LigPrep against COX2 was performed in triplicate and used as the model experiment to assess the similarity of agent generated compounds to known actives and decoys. The top 150 compounds (based on total score) from each replicate were pooled and duplicates removed, resulting in 298/450 unique compounds, which amounts to about 33% overlap between the individual replicates. The Tanimoto similarities were calculated for each top generated compound compared to each known active (40 total) or decoy (1200 total), as provided in the DEKOIS 2.0 dataset, respectively. [26] The highest Tanimoto similarities were kept, resulting in distributions describing the maximum resemblance of a top generated compound (based on total score) to a known active or known decoy (Fig. 7). The prior was trained on the ChEMBL dataset and contains 33/40 and 1/1200 of the known actives and known decoys, respectively. While no actives were recovered, it is evident that the agent generated compounds with notable structural similarity to known actives as measured by a Tanimoto similarity > 0.7 (Fig. 7a). It follows from Fig. 6b that many of the top compounds contain the sulfonamide moiety which is present in the approved drugs, Celecoxib and Valdecoxib [60, 61]. The maximally similar generated compound displays a Tanimoto similarity of 0.937 and shares both the sulfonamide moiety and the *cis*-stilbene scaffold with its corresponding known active (Fig. 7a). In contrast, the highest Tanimoto similarity observed for the most similar decoy to a top generated compound is 0.631 (Fig. 7b). While the scaffold is shared, the vital sulfonamide moiety is not present. Moreover, the decoy ligands set (1200 total) is 30 × larger than the active ligands set (40 total). If the agent policy was not meaningfully updated during the generative process, the likelihood of observing a high Tanimoto similarity (> 0.7) in the decoys set should be increased. Thus, the integration of DockStream with REINVENT facilitates agent convergence to relevant chemical space and the absence of actives recovered does not preclude the generated compounds from being true actives and potentially even more potent. Overall, the absence of Tanimoto similarities above 0.631 amongst the decoys and the presence of Tanimoto similarities > 0.7 amongst the actives suggests the agent is biased towards generating compounds that are more likely to be true actives.

### 3D structural awareness

**Fig. 8:**
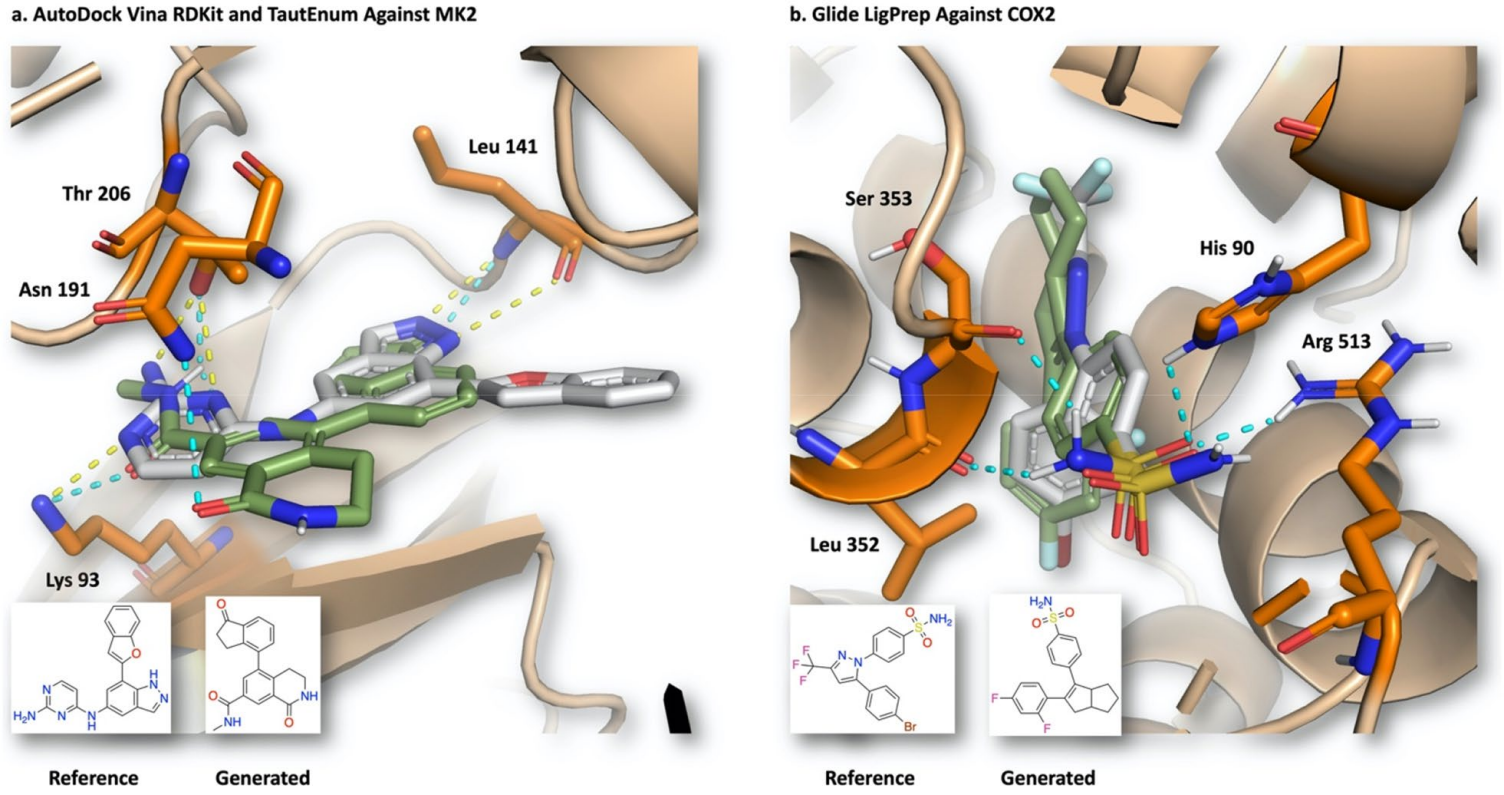
Binding poses of generated compounds from selected experiments shown in Fig. 5 (see Additional file 1: Fig. S34 for all other experiments). **a** AutoDock Vina with RDKit and TautEnum against MK2 (**PDB ID: 3KC3**) **b** Glide with LigPrep against COX2 (**PDB ID: 1CX2**). “Reference” (gray) is the co-crystallized ligand and its interactions are shown as yellow dotted lines. “Generated” (green) is a selected compound from the top 10 compounds (based on total score) and its interactions are shown as turquoise dotted lines. Note: The sulfonamide moiety of the reference ligand, as provided in the crystal structure, should be rotated 180° such that the NH_2_ hydrogens are pointed towards Leu 352 and Ser 353 rather than His 90 and Arg 513

While generated compounds can satisfy the scoring function almost perfectly, their usefulness strongly depends on the plausibility of their binding poses, as commonly assessed in virtual screening campaigns. To further investigate agent 3D structural awareness, the predicted binding poses of one ligand from the top 10 compounds (based on total score) produced by the experiments shown in Fig. 4 were analyzed (Fig. 8). Docking against MK2, a serine and threonine kinase target for anti-inflammation, was performed using AutoDock Vina with RDKit and TautEnum. Structure–activity relationship (SAR) studies demonstrated interactions between the co-crystallized ligand and residues Lys 93 and Thr 206 are vital for potency [63]. The generated ligand retains these interactions and is predicted to exploit an additional hydrogen bonding interaction with Asn 191 (Fig. 8a). The overlap between the reference and generated ligand suggests the latter as a plausible binding pose and represents a solution obtained using only free software. Similarly, docking against COX2, a target for anti-inflammation, was performed using Glide with LigPrep [60, 61]. SAR studies demonstrated the *cis*-stilbene motif and the phenylsulfonamide moiety are paramount for potency and selectivity over COX1, respectively. The latter is attributed to the smaller residue Val 523 in COX2 in place of Ile 523 in COX1 facilitating easier access to the binding cavity [61]. The generated ligand retains the *cis*-stilbene backbone and the sulfonamide group, which is predicted to form hydrogen bond interactions with Leu 352, Ser 353, His 90, and Arg 513 (Fig. 8b). Notably, approved drugs against COX2 include Celecoxib and Valdecoxib which also contain the aforementioned structural features [61]. Crucially, the predicted binding pose for the de novo compound overlaps significantly with the co-crystallized ligand which is another selective COX2 inhibitor (SC-558), yielding greater confidence in its plausibility [60, 61]. The same analysis was conducted for the GOLD, Hybrid, and rDock experiments shown in Fig. 4. Docking against HDAC2, a histone deacetylase, was performed using GOLD with RDKit and TautEnum. SAR studies demonstrated the interaction with zinc and large bulky groups that extend deep into the ‘foot pocket’ are important for potency [64]. The generated ligands retain interactions with zinc and Gly 154 and overlap significantly with the co-crystallized ligand (see Additional file 1: Fig. S34i). In another experiment, we used Hybrid with LigPrep as a docking component to design compounds against retinoid X receptor alpha (RXRα), a vital component in nuclear receptors. The co-crystallized ligand, CD3254 is an agonist and binds by modulating the H12 helix into its ‘active’ conformation [65]. While the generated ligands do not retain these interactions, their predicted binding poses are not in conflict with the required conformation of the H12 helix. On the other hand, a common interaction with Arg 316 which is formed by endogenous ligands such as 9-*cis* retinoic acid is retained by the generated ligands, although there is some precedent that its role to facilitate binding is not essential (see Additional file 1: Fig. S34k) [66]. Finally, docking against thymidylate synthase (TS), responsible for converting deoxyuridine monophosphate (dUMP) into deoxythymidine monophosphate (dTMP) was performed using rDock with RDKit and TautEnum. The co-crystallized ligand is Tomudex, an approved drug which interacts with dUMP via stacking interactions and the quinazoline ring forms the only hydrogen bond interactions with Asp 218 and Gly 222 (see Additional file 1: Fig. S34o) which are retained by the generated ligands [67]. Overall, all REINVENT-DockStream experiments generated compounds that retain vital interactions and display excellent agreement with reference ligand binding poses, suggesting the agent learns implicitly 3D structural information (see Additional file 1: Table S1 for all REINVENT-DockStream experiments and Fig. S34 for all selected binding poses).

### Steering chemical space exploration to diverse local minima

**Fig. 9:**
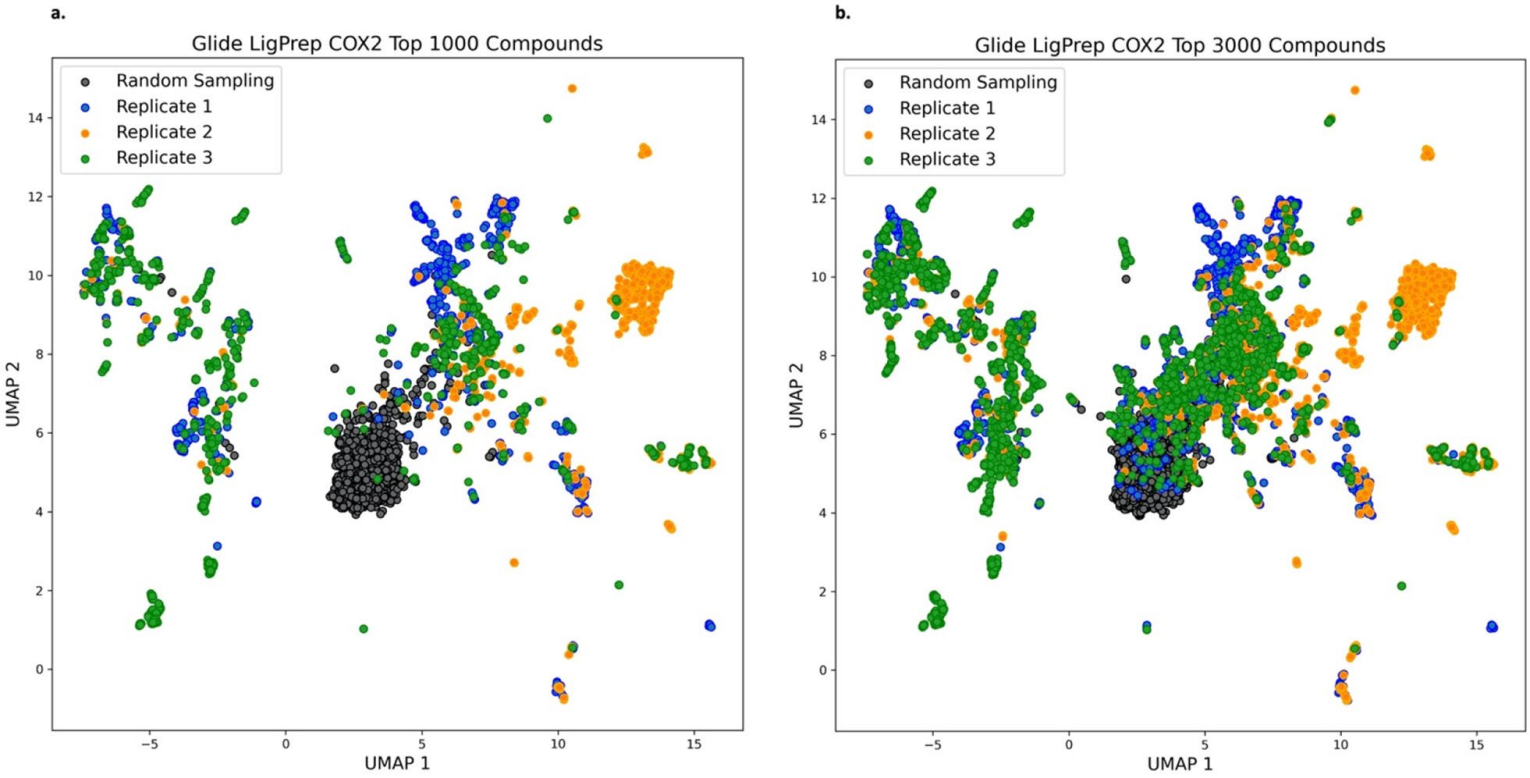
Uniform Manifold Approximation and Projection (UMAP) dimensionality reduction to illustrate chemical space coverage of triplicate Glide with LigPrep REINVENT-DockStream experiments against COX2 (**PDB ID: 1CX2**). Morgan fingerprints (radius 3, 1024 bits) for the top 1000 and 3000 compounds (based on total score) for each replicate were extracted to illustrate topology features. Chemical space coverage is stochastic in nature and thus, multiple replicates are used in practice to optimize output diversity. “Random Sampling” refers to sampling compounds from the prior generative model whose policy has not been focused on any task. **a** Top 1000 (based on total score) compounds generated by the agent in each triplicate experiment **b** Top 3000 (based on total score) compounds generated by the agent in each triplicate experiment. Some overlap between replicate experiments is observed, demonstrating steering of chemical space exploration to similar local minima

The diversity and convergence of REINVENT-DockStream was investigated on a larger scale. Uniform Manifold Approximation and Projection (UMAP) was used as a dimensionality reduction technique to visualize the span of chemical space occupied by generated compounds compared to random sampling by the prior which has not been focused on any task (Fig. 9) [68]. REINVENT using Glide with LigPrep against COX2 was performed in triplicate and visualized by reducing the Morgan fingerprints (radius 3, 1024 bits) of the top 1000 and 3000 proposed compounds to 2D [69]. It is evident that the random sampling of molecules from the ChEMBL dataset occupies a single cluster (Fig. 9). In contrast, agent perceived desirable compounds occupy a much more diverse chemical space, forming numerous clusters representing different local minima. Importantly, this necessitates the compounds to be diverse, further supporting the application of a DF in molecular generation and demonstrating balance between agent exploration and exploitation. Interestingly, the chemical space spanned by the top compounds from the Glide REINVENT triplicate experiments overlap significantly. The agent is initialized at a random chemical space and is iteratively updated to satisfy the pre-defined scoring function. The UMAP results suggest that irrespective of the chemical space starting point and the stochasticity associated with Glide and the conditional probabilities of token sampling, iterative optimization can lead to convergence. These results demonstrate steering of chemical space exploration via RL and suggest that while replicate experiments yield a more complete coverage of chemical space (deemed favourable by the agent), single experiments may not drastically compromise agent exploration.

## Discussion

In this work, we present DockStream, a molecular docking wrapper providing facile access to a collection of ligand embedders and docking backends. The capabilities of the platform are extended by the benchmarking and analysis workflow which automates molecular docking and post hoc analysis. The use of DockStream as a stand-alone docking tool was demonstrated by reproducing and extending the DEKOIS 2.0 dataset with additional docking backends (Hybrid and rDock) and ligand embedders (Corina and RDKit). DockStream facilitates large-scale automation of molecular docking and supports various computational chemistry software suites. The supported analysis modes can enhance VS endeavours by expediting the identification of a suitable docking configuration. It was unsurprising that the DEKOIS 2.0 benchmarking demonstrated that docking can be particularly sensitive to the receptor and docking parameters, whereby small modifications in the protocol can have an enormous impact on enrichment. DockStream also outputs the binding poses, providing a convenient intermediate for downstream computational experiments such as re-scoring or more accurate methods to assess binding free energy. To this end, the extensive capabilities of DockStream ligand enumeration provides excellent compatibility with the aforementioned techniques which often require numerous conformational representations [70].

The integration of DockStream with the de novo design platform, REINVENT, enhances its generative capabilities beyond standard QSAR models which often suffer from limited domain applicability [11, 12]. In particular, one is often interested in focusing on either agent chemical space exploration or exploitation, which are both supported in REINVENT. While QSAR models can perform well in the latter scenario provided the predictive model was trained on similar compounds (often belonging to the same series), extrapolating to new chemical space will likely be inaccurate. Molecular docking is chemical space agnostic and provides a more generalized solution to incorporating structural information during the generative process. However, docking will greatly increase the computational costs relative to trained QSAR models, warranting consideration over the quality of compounds generated. To assess the performance of REINVENT-DockStream, 15 different targets from the DEKOIS 2.0 dataset along with their corresponding docking configurations were selected [26]. We emphasize the choice of docking configurations that display enrichment and offer distinction between ‘good’ docking scores, providing an unambiguous endpoint for docking score optimization and increases the likelihood of generated compounds being true actives. We show that REINVENT-DockStream optimizes docking scores across all docking backends while maintaining “drug-likeliness” as enforced by the QED score [57]. Furthermore, molecular weight was incorporated in the scoring function which helped circumvent artificially high docking scores displayed in relatively large molecules, owing to the sheer number of interactions possible. Large molecules may also be more prone to entropically driven binding, causing off-target effects [52]. Thus, generating compounds that conform to a reasonable size increases the overall quality of the results. Finally, the use of a diversity filter maintains agent exploration as demonstrated by the number of unique SMILES proposed [4].

In order to elucidate the balance between agent exploration and exploitation in REINVENT-DockStream experiments, the average linkage similarity between epochs (Tanimoto) was calculated [62]. The results demonstrate increased intra-batch similarity relative to surrounding epochs, indicating agent sampling from similar chemical space within a given epoch. Moreover, as agent training proceeds, there is a clear transition to greater similarity values at later epochs, paralleling the transition from agent exploration to exploitation. To further assess generated compounds from agent exploitation, the generated top compounds (based on total score) were compared to known actives and known decoys as provided in the DEKOIS 2.0 dataset [26]. While the known actives were not recovered, the top compounds show a clear enrichment in the Tanimoto similarities over the known decoys. The results demonstrate the agent is steered to chemical space similar to that of experimentally validated active compounds. Furthermore, the quality of generated compounds was analyzed by comparing their binding poses relative to known binders. We show that generated compounds retain vital interactions in the binding cavity and exploit new ones. Crucially, the binding poses overlap with known binders, increasing confidence in the prediction. Moreover, we show that 3D structural information is implicitly incorporated during the generative process by exploiting structural motifs and moieties that conform to subtle changes in the binding cavity. Finally, the stochasticity of REINVENT-DockStream was investigated by visualizing the chemical space spanned by the top generated molecules using Uniform Manifold Approximation and Projection (UMAP) as a dimensionality reduction technique [68]. Compared to random sampling, agent generated compounds form clusters over a diverse area and generally converge over replicate runs.

Further work will focus on expanding the capabilities of DockStream to address limitations in the reliability of docking scores. Currently, the output binding poses can only be triaged manually but provide an invaluable database for further structure-based methods. In particular, negative image-based rescoring (R-NiB) has been shown to enhance enrichment in terms of activity [71]. Other possible enhancements include an explicit handling of ligand internal strain energy which is typically not well captured by docking algorithms. It has been shown that proper consideration can enhance docking enrichment and limit the number of false positives [72]. DockStream will receive continued support to ensure all supported backends are up to date, such as the integration of the recently reported AutoDock Vina 1.2.0 functionalities [73]. The DockStream codebase is provided at https://github.com/MolecularAI/DockStream. An additional repository with executable tutorials on supported features and workflows is provided at https://github.com/MolecularAI/DockStreamCommunity.

## Conclusions

Generative models have been successfully applied to de novo design, generating compounds which satisfy a wide variety of properties. The integration of QSAR models aim to incorporate structural information in the generative process. However, these models can suffer from limited applicability domains. Consequently, extrapolating out of sample can be unreliable and potentially misinform the generative agent especially when chemical space exploration is desired. We built upon recent work that incorporates molecular docking in place of QSAR models for better generalization. DockStream is presented as a molecular docking wrapper which provides access to a collection of ligand embedders and docking backends. The use of DockStream as a stand-alone docking tool was demonstrated by large-scale docking efforts for a wide variety of targets and showcasing the automation of docking execution and post hoc analysis via the benchmarking and analysis workflow. The results show that it is beneficial to integrate several ligand embedders and docking backends in DockStream, so as to find a productive docking configuration for diverse end applications. The integration of DockStream with the recently published de novo design platform, REINVENT, demonstrates docking score optimization across all supported docking backends. The generated compounds retain vital interactions in the binding cavity and exploit new interactions. Extensive agreement with binding poses of known binders increases the confidence of the generated compounds and demonstrates the value of using docking to implicitly inform the generative process of 3D structural information. DockStream has the potential to be especially impactful in REINVENT experiments aiming at exploring novel chemotypes by providing a chemical space agnostic component that helps the agent learn to exploit the shape and intermolecular interactions of binding cavities.

## Methods

### Target preparation

Receptor crystal structures were obtained from the Protein Data Bank (PDB) with corresponding PDB IDs as specified in the DEKOIS 2.0 dataset (except SIRT2 which used PDB ID 5Y0Z so as to provide a reference ligand) [26, 74]. The PDB structures were processed by removing redundant chains possessing duplicate ligands, co-factors, and ions. All water molecules were also removed. The configurations for further processing depended on the docking backend to be used. For docking with AutoDock Vina, Glide, or rDock, the Protein Preparation Wizard in Maestro (release 2019–4) was used [75, 76]. The PDB structures were pre-processed with default parameters, followed by PROPKA hydrogen bond network optimization at pH 7.4, and minimized using the OPSL3e force-field [77]. Alternatively, for docking with Hybrid or GOLD, PDBFixer (Conda package version 1.7) was used to remove heterogens and add missing heavy atoms and hydrogens [78]. All processed receptor structures were saved as PDB files and used as is for grid generation.

### Receptor grid generation

Receptor grids were generated using the processed PDB structures and followed different docking specific configurations. For AutoDock Vina, Open Babel (Conda package version 3.1.1) was used to convert the PDB file into PDBQT format and coordinates for the binding cavity were defined using the reference ligand [79]. The grid box size was 15 X 15 X 15 Å. For Glide, the reference ligand was used to define the binding cavity in Receptor Grid Generation in Maestro [80]. Default parameters were used, specifying a grid box size of 20 X 20 X 20 Å to generate the corresponding ZIP grid file. For GOLD, the BindingSiteFromLigand class in the Docking API (release 2020.0.1 CSD) using the reference ligand with the distance parameter set to 10 Å was used to generate the grid [38, 81]. For Hybrid, the OEMakeReceptor method in the OEDocking API (release 3.0.8) using the reference ligand was used to generate the OEB grid [39]. For rDock, the rbcavity method (version 2013.1) using the reference ligand (in cases where an atom parsing error occurred, the reference ligand was first processed in the Protein Preparation Wizard) with radius set to 5 Å centered on each atom was used to generate the updated PRM grid file. The output from each of these configurations was used as is for docking.

### Ligand preparation

The ligands SMILES for the actives and decoys sets were downloaded from the DEKOIS 2.0 web server [26]. The ligands were processed following different configurations depending on the ligand embedder used. For Corina with TautEnum (tool to enumerate tautomers and protonation states, version 2.0.0), default parameters were used [27–29, 54, 55]. For LigPrep, default parameters were used except for Epik, for which the target pH was set to 7.0 with a tolerated range of ± 2.0 [30]. The ligand energies were minimized using the OPSL3e force-field [77]. For RDKit (version 2020.09.3) with TautEnum, default parameters were used except for the number of maximum iterations, which was set to 600 (for Universal Force-Field, UFF) to ensure convergence for all compounds [82]. All prepared ligands were saved as SDF files and also stored internally in DockStream as RDKit molecules for subsequent docking.

### Docking

The entire DEKOIS 2.0 dataset was docked using AutoDock Vina, Glide, GOLD, Hybrid, and rDock using ligands embedded by Corina with TautEnum, LigPrep, and RDKit with TautEnum, resulting in 15 docking runs per protein target and a total of 1215 docking experiments amounting to over 1,506,600 ligands docked [27–30, 33–38, 40, 41, 54, 55]. Default docking parameters were used unless specified as follows: AutoDock Vina and rDock were configured to return 2 and 12 poses, respectively. GOLD used the ChemPLP scoring function and was allowed up to 10 docking attempts with autoscale set to 0.25. Generally, the active and decoy ligands sets were comprised of 40 and 1200 ligands, respectively (39 actives and 1170 decoys for SARS-HCoV and 1199 decoys for TS). Only the best tautomer or ionization state (if applicable) per ligand as assessed by its docking score was kept. In some cases, ligands failed to be embedded or docked, which are attributed to ligand embedder failure or the docking backends’ inability to find a good pose. In addition, AutoDock Vina and GOLD failed to dock all ligands for 23/81 targets (ACE, ACE2, ADAM17, COX1, COX2, CYP2A6, EPHB4, FXA, HDAC2, HDAC8, HIV1PR, HIV1RT, KIF11, MMP2, NA, PDE4B, PDE5, PNP, QPCT, SARS-HCoV, SIRT2, Thrombin, and TK) and 4/81 targets (11betaHSD1, ALR2, HIV1PR, and INHA), respectively. In the original DEKOIS 2.0 work, this was resolved via manual expansion of the binding cavity which was not explored in this work [26].

### Average linkage similarity (Tanimoto)

The average linkage similarity between epochs (Tanimoto) was calculated for each REINVENT-DockStream experiment for every 5 epochs instead of every epoch to reduce the computational time while maintaining good interpretability. The average linkage similarity, L, is given by Eq. 5 [62]:

5 $$L\left( {e_{1} , e_{2} } \right) = \frac{1}{{N_{1} N_{2} }}\mathop \sum \limits_{i}^{{N_{1} }} \mathop \sum \limits_{j}^{{N_{2} }} T\left( {x_{{e_{1} i}} ,x_{{e_{2} j}} } \right)$$

where e_1_ and e_2_ are epochs 1 and 2, respectively, N_1_ and N_2_ are the number of molecules in epochs 1 and 2, respectively, and T is the Tanimoto similarity between molecules, denoted $x$. The result of Eq. 5 is a 200 × 200 heatmap (as the average linkage similarity was calculated for every 5 epochs) for every REINVENT-DockStream experiment (see Additional file 1: Figs. S35–51).

## Supplementary Information

**Additional file 1**. Zipped archive of the JSON files used to generate the study.

## Abbreviations

MPO: Multi-parameter optimization
RNN: Recurrent neural network
VAE: Variational autoencoder
GAN: Generative adversarial network
GNN: Graph neural network
RL: Reinforcement learning
QSAR: Quantitative structure–activity relationship
VS: Virtual screening
SBDD: Structure-based drug discovery
3D: 3-Dimensional
RMSD: Root-mean-square deviation
pROC: Logarithmic receiver operating characteristic
AUC: Area under the curve
LOQ: Limit of quantification
NLP: Natural language processing
GRU: Gated recurrent unit
TPSA: Topological polar surface area
MW: Molecular weight
HBD: Number of hydrogen bond donors
DF: Diversity filter
PAINS: Pan-assay interference compounds
QED: Quantitative Estimate of Druglikeness
UMAP: Uniform Manifold Approximation and Projection
R-NiB: Negative image-based rescoring
